# Association between diabetes duration and 1‐year prognosis of stroke: A national registry study

**DOI:** 10.1002/brb3.2725

**Published:** 2022-08-08

**Authors:** Yanli Zhang, Aoming Jin, Xia Meng, Mengxing Wang, Hao Li, Yuesong Pan, Yongjun Wang

**Affiliations:** ^1^ Department of Neurology, Beijing Tiantan Hospital Capital Medical University Beijing China; ^2^ China National Clinical Research Center for Neurological Diseases Beijing China; ^3^ Advanced Innovation Center for Human Brain Protection Capital Medical University Beijing China; ^4^ Center for Excellence in Brain Science and Intelligence Technology Chinese Academy of Sciences Shanghai China

**Keywords:** clinical outcome, diabetes duration, prognosis, stroke recurrence

## Abstract

**Background and purpose:**

Diabetes mellitus is a strong independent risk factor for stroke recurrence. However, the association between diabetes duration and the prognosis of stroke remains uncertain. We aimed to characterize whether an association exists between diabetes duration and stroke outcomes in patients with ischemic stroke or transient ischemic attack (TIA).

**Methods:**

Between 2015 and 2018, 14,674 patients with ischemic stroke or TIA within 7 days and older than 18 years from the Third China National Stroke Registry (CNSR‐III) were included in this analysis. Diabetes duration at baseline was collected by face‐to‐face interviews and further categorized into groups of without diabetes, diabetes < 4, 4 to <8 and ≥8 years. The association between diabetes duration and clinical outcomes, including stroke recurrence, poor function outcome (modified Rankin Scale score of 3–6), and all‐cause mortality at the 1‐year follow‐up after stroke onset, was evaluated by a multivariable Cox proportional hazard regression model, competing risk model and logistic regression model with adjustment for demographic and clinical features.

**Results:**

Among the 14,674 patients included, the average age was 62.0 years, and 68.5% were male. There were 1419 (9.7%) patients who had stroke recurrence, 1912 (13.0%) who had poor function outcome, and 478 (3.3%) who had all‐cause mortality at the 1‐year follow‐up. After adjusting for potential covariates, a diabetes duration ≥8 years was associated with an increased risk of 1‐year stroke recurrence (adjusted hazards ratio [HR], 1.31; 95% CI, 1.05–1.64; *p *= .02) in comparison to those without Diabetes mellitus. Using a competing risk regression model, a diabetes duration ≥8 years was a significant risk factor for stroke recurrence (HR, 1.31; 95% CI, 1.12–1.53). In contrast, there was no significant association between diabetes duration < 4, 4 to <8 years and clinical outcomes.

**Conclusions:**

Long‐term diabetes duration (≥8 years), but not short‐term diabetes duration, was associated with an increased risk of 1‐year stroke recurrence in patients with ischemic stroke or TIA.

## Graphical abstract

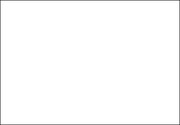

## 1 INTRODUCTION

Stroke is a primary cause of mortality and disability throughout the world (GBD 2016 Neurology Collaborators, 2019). Approximately 11% of the population may experience stroke recurrence within a year after their first stroke (Mohan et al., 2011). Moreover, it is estimated that one third of all stroke patients have diabetes mellitus (DM). Growing evidence has demonstrated that DM is an independent predictor of stroke, especially ischemic stroke (Alloubani et al., 2018; Bailey et al., 2020; Homoud et al., 2020). Several clinical studies reported that diabetes duration had a greater influence on prognosis than diabetes itself and that diabetes‐related complications increased with disease progression (Reinke et al., 2021) Previous studies (Kosiborod et al., 2018; Noh et al., 2017; Sarwar et al., 2010; Verma et al., 2019) have shown that long‐term diabetes duration is associated with a higher risk of major adverse cardiovascular events. A recent study elaborated that a longer duration of DM was independently associated with intracranial atherosclerotic stenosis (Fujiyoshi et al., 2020), which might accelerate plaque formation and lead to the occurrence of stroke. However, existing data are limited to our knowledge regarding the relationship of different diabetes durations with the clinical prognosis of stroke.

Therefore, this study aimed to evaluate whether an association exists between diabetes duration and the risk of 1‐year prognosis of stroke in ischemic stroke or transient ischemic attack (TIA) patients.

## 2 METHODS

### 2.1 Study design and participants

We derived data from the Third China National Stroke Registry (CNSR‐III) trial. The CNSR‐III was a large‐scale nationwide, hospital‐based, prospective cohort study that recruited 15,166 consecutive patients to evaluate the etiology, imaging, and biological markers for the prognosis of ischemic stroke or TIA between August 2015 and March 2018 from 201 study sites covering 26 provinces and municipalities in China. Patients within 7 days of the onset of symptoms and older than 18 years were enrolled in this study. More detailed information about the rationale, design, follow‐up procedures, and outcome classifications of the CNSR‐III study has been published elsewhere (M. Wang et al., 2019). The protocol of the CNSR‐III study was approved by the ethics committee of Beijing Tiantan Hospital. Written informed consent was obtained from all patients or legally authorized representatives before entering into the study.

### 2.2 Baseline data collection

Trained research coordinators of CNSR‐III collected baseline information following a standard data collection protocol developed by the steering committee. Patients were contacted through face‐to‐face interviews within 24 h after admission. Baseline information included demographics, medical history, stroke subtype defined by the Trial of Org 10172 in Acute Stroke Treatment (TOAST), prestroke modified Rankin Scale (mRS), National Institutes of Health Stroke Scale (NIHSS) score, laboratory examination, and medication use at discharge. Medical history included prior stroke, hypertension, dyslipidemia, atrial fibrillation, and coronary artery disease. Stroke subtypes included large artery atherosclerosis, cardioembolism, small‐artery occlusion, other determined ethology, and undetermined ethology (Adams et al., 1993). The estimated glomerular filtration rate (eGFR) was calculated by serum creatinine levels using modified equations from the Chronic Kidney Disease Epidemiology Collaboration (CKD‐EPI) with an adjusted coefficient of 1.1 for the Chinese population (X. Wang et al., 2014). An eGFR ≥90 ml/min/1.73 m^2^ indicated normal renal function.

### 2.3 Evaluation of diabetes duration

Data with respect to history of DM and diabetes duration were collected at baseline. Previously diagnosed DM was defined as a self‐reported previous medical diagnosis at recruitment or the use of any antidiabetic medication (Herrington et al., 2018). In particular, patients with diagnosed DM during hospitalization were categorized as new onset DM. We defined the diabetes duration as the number of years between the previously diagnosed DM and symptom onset of the index ischemic event (ischemic stroke or TIA). Based on our data and previous research, the patients were further classified into four groups according to diabetes duration: without DM, diabetes duration <4 years, diabetes duration 4 to <8 years, and diabetes duration ≥8 years (Hu et al., 2021).

### 2.4 Outcome assessment

Follow‐up was performed by telephone interview. Data were collected by trained research coordinators who were blinded to patient baseline status based on a standardized interview protocol. Outcome data at 1 year after stroke onset included stroke recurrence, poor function outcome, and all‐cause mortality, which were evaluated by the independent endpoint judgment committee according to the endpoint information. During the follow‐up periods, all‐cause mortality and stroke recurrence were recorded, and mortality was certified by the attending hospital or the local citizen registry. Stroke recurrence was defined as an aggravated primary neurologic deficit in which the NIHSS score increased by 4 points or above, a new neurological deficit lasting more than 24 hours, or rehospitalization with a diagnosis of ischemic stroke, intracerebral hemorrhage, or subarachnoid hemorrhage (WHO, 1989). A poor function outcome was defined as an mRS score of 3–6.

### 2.5 Statistical analysis

Baseline variables were presented categorical variables as percentages and continuous variables as means with standard deviations (SDs). The univariable analyses of baseline variables among different diabetes duration groups were compared using the *χ* ^2^ test for categorical variables and one‐way ANOVA or Kruskal‒Wallis test for continuous variables.

The Cox proportional hazard regression model was used to assess the association of diabetes duration with stroke recurrence and all‐cause mortality from symptom onset by calculating hazard ratios (HRs) and 95% confidence intervals (CIs). The proportional hazards assumption for the Cox model was tested by adding a time‐dependent covariate with the interaction of the diabetes duration groups and a logarithmic function of survival time in the model. The multivariable logistic regression model was used for assessment of variables that were associated with poor function outcome by calculating odds ratios (OR) and 95% CI. For each outcome, we performed three models. Model 1 adjusted for only age and sex. Model 2 adjusted for demographics, medical history, index event, stroke severity and subtype on admission, and medication at discharge. Model 3 adjusted for covariates in model 2 plus hemoglobin A1c (HbA1c) on admission with inverse probability‐weighted analysis for missing data to estimate whether control of blood glucose level (estimated by HbA1c) changed the association between diabetes duration and prognosis of stroke. Accounting for competing risks was necessary because for stroke recurrence, specific competing events could occur, which prevented the occurrence of the endpoint of interest. We further used the Fine and Gray regression model for a subdistribution function treating all‐cause mortality of the patients as a competing event, and Nelson‒Aalen cumulative risk curves were calculated and then compared by Gray's test. We also employed restricted cubic spline analysis to test for potential nonlinear relationships between diabetes duration and stroke outcomes.

All data were analyzed with SAS version 9.4 statistical software (SAS Institute Inc, Cary, NC). All tests were two‐sided, and a *p*‐value less than .05 was considered statistically significant.

## 3 RESULTS

### 3.1 Baseline characteristics

**FIGURE 1:**
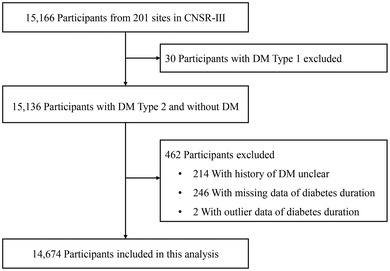
Study flowchart of the participant selection. Abbreviations: CNSR‐III, Third China National Stroke Registry; DM, diabetes mellitus

A total of 15,166 ischemic stroke or TIA patients were enrolled in the CNSR‐III, of whom 30 were excluded due to type 1 DM on admission. In addition, patients with unclear history of DM (*n* = 214), without data of diabetes duration (*n* = 246), or with outlier data of diabetes duration (*n* = 2) were also excluded. After exclusion of these patients, a total of 14,674 patients were included in the final analysis (Figure 1). Among all 14,674 patients included, the mean age of the study patients was 62.2 ± 11.3 years, and 10,057 (68.5%) patients were male. There were 6205 (42.3%) patients with missing data for HbA1c. A total of 1150 (7.8%) patients had an index event of TIA, and 13,524 (92.2%) patients had ischemic stroke. The baseline demographic and clinical characteristics of patients are shown in Table 1.

**TABLE 1:** Baseline characteristics of patients in different diabetes duration groups

	Total	Patients without DM	Patients with DM, by duration			*p*‐Value
			<4 years	4 to <8 years	≥8 years	
Number	14,674	10,086	2240	797	1551	
Age, years, mean ± SD	62.2 ± 11.3	62.0 ± 11.7	61.3 ± 10.8	62.2 ± 10.1	64.6 ± 9.8	<.001
Male, *n* (%)	10,057 (68.5)	7088 (70.3)	1511 (67.5)	493 (61.9)	965 (62.2)	<.001
Current smoking, *n* (%)	4615 (31.5)	3322 (32.9)	707 (31.6)	227 (28.5)	359 (23.2)	<.001
Regular drinking, *n* (%)	2391 (16.3)	1707 (16.9)	376 (16.8)	114 (14.3)	194 (12.5)	<.001
Medical history, *n* (%)						
Prior stroke	3247 (22.1)	2089 (20.7)	485 (21.7)	230 (28.9)	443 (28.6)	<.001
Hypertension	9165 (62.5)	5940 (58.9)	1519 (67.8)	591 (74.2)	1115 (71.9)	<.001
Dyslipidemia	1135 (7.7)	614 (6.1)	203 (9.1)	112 (14.1)	206 (13.3)	<.001
Atrial fibrillation	985 (6.7)	753 (7.5)	116 (5.2)	38 (4.8)	78 (5.0)	<.001
Coronary artery disease	1543 (10.5)	926 (9.2)	229 (10.2)	123 (15.4)	365 (17.1)	<.001
Index event, *n* (%)						<.001
TIA	1150 (7.8)	850 (8.4)	162 (7.2)	51 (6.4)	87 (5.6)	
Ischemic stroke	13,524 (92.2)	9236 (91.6)	2078 (92.8)	746 (93.6)	1464 (94.4)	
TOAST, *n* (%)						<.001
LAA	3733 (25.4)	2429 (24.1)	631 (28.2)	227 (28.5)	446 (28.8)	
CE	891 (6.1)	667 (6.6)	109 (4.9)	37 (4.6)	78 (5.0)	
SAO	3062 (20.9)	2057 (20.4)	454 (20.3)	202 (25.4)	349 (22.5)	
Other determined cause	178 (1.2)	127 (1.3)	26 (1.2)	11 (1.4)	14 (0.9)	
Undetermined cause	6810 (46.4)	4806 (47.7)	1020 (45.5)	320 (40.2)	664 (42.8)	
Prestroke mRS, *n* (%)						.99
0–2	14,053 (95.8)	9663 (95.8)	2143 (95.7)	763 (95.7)	1484 (95.7)	
3–5	621 (4.2)	423 (4.2)	97 (4.3)	34 (4.3)	67 (4.3)	
NIHSS on admission, *n* (%)						.001
<5	9610 (65.5)	6636 (65.8)	1449 (64.7)	533 (66.9)	992 (64.0)	
5–15	4661 (31.8)	3137 (31.1)	745 (33.3)	253 (31.7)	526 (33.9)	
>15	403 (2.8)	313 (3.1)	46 (2.1)	11 (1.4)	33 (2.1)	
Laboratory examination						
eGFR*≥90(ml/min/1.73 m^2^), *n* (%)	11,734 (80.0)	8064 (80.0)	1847 (82.5)	640 (80.3)	1183 (76.3)	<.001
SBP, mm Hg, mean ± SD	150.0 ± 22.1	149.2 ± 22.1	152.5 ± 22.3	150.3 ± 21.0	151.8 ± 21.5	<.001
DBP, mm Hg, mean ± SD	87.3 ± 13.1	87.4 ± 13.3	88.9 ± 13.1	86.6 ± 12.5	85.20 ± 11.7	<.001
BMI, kg/m^2^, mean ± SD	24.7 ± 3.3	24.4 ± 3.3	25.5 ± 3.3	25.3 ± 3.3	24.9 ± 3.2	<.001
HbA1c, mmol/L, mean ± SD	6.47 ± 1.78	5.63 ± 0.99	7.48 ± 1.75	8.16 ± 1.89	8.57 ± 1.89	<.001
CHO, mmol/L, mean ± SD	4.12 ± 1.22	4.09 ± 1.19	4.22 ± 1.28	4.14 ± 1.32	4.16 ± 1.29	.002
TG, mmol/L, mean ± SD	1.59 ± 0.90	1.49 ± 0.83	1.79 ± 1.04	1.80 ± 0.99	1.75 ± 0.98	<.001
LDL‐C, mmol/L, mean ± SD	2.45 ± 1.07	2.43 ± 1.04	2.50 ± 1.10	2.42 ± 1.15	2.47 ± 1.15	.12
HDL‐C, mmol/L, mean ± SD	0.97 ± 0.30	0.99 ± 0.31	0.92 ± 0.28	0.91 ± 0.29	0.92 ± 0.28	<.001
Medication at discharge						
Antihypertensive	7140 (48.8)	4639 (46.1)	1228 (54.9)	414 (52.1)	859 (55.6)	<.001
Lipid‐lowering	13,390 (91.5)	9141 (90.8)	2088 (93.3)	735 (92.5)	1426 (92.3)	<.001
Antiplatelet	13,273 (90.7)	9062 (90.0)	2060 (92.1)	739 (93.0)	1412 (91.4)	.001

Abbreviations: BMI, body mass index; CE, cardioembolism; CHO, cholesterol; DBP, diastolic blood pressure; DM, diabetes mellitus; eGFR, estimated glomerular filtration rate; HDL‐C, high‐density lipoprotein cholesterol; HbA1c, hemoglobin A1c; LAA, large‐artery atherosclerosis; LDL‐C, low‐density lipoprotein cholesterol; mRS, modified Rankin Scale; NIHSS, National Institutes of Health stroke scale; SAO, small‐artery occlusion; SBP, systolic blood pressure; SD, standard deviation; TIA, transient ischemic attack; TOAST, stroke subtype defined by the Trial of Org 10172 in Acute Stroke Treatment classification; TG, triglycerides.

* Values of eGFR were calculated by serum creatinine using the equations from the Chronic Kidney Disease Epidemiology Collaboration.

According to the diabetes duration, all patients were divided into four groups: 10,086 (68.7%) patients without DM, 2240 (15.3%) with duration <4 years, 797 (5.4%) with duration 4 to <8 years, and 1551 (10.6%) with duration ≥8 years. Patients with long‐term diabetes duration had a higher level of HbA1c and triglycerides, history of prior stroke, hypertension and dyslipidemia, and lower high‐density lipoprotein levels at admission. These patients were less likely to be current smokers and regular drinkers, had the subtypes of ischemic stroke and large artery atherosclerosis, and received more antihypertensive, lipid‐lowering, antiplatelet agents at discharge.

### 3.2 Association of diabetes duration with clinical outcomes

Among 14,674 patients with ischemic stroke or TIA, stroke recurrence occurred in 1419 (9.7%), poor function outcome in 1912 (13.0%), and all‐cause mortality in 478 (3.3%) patients at the 1‐year follow‐up.

**FIGURE 2:**
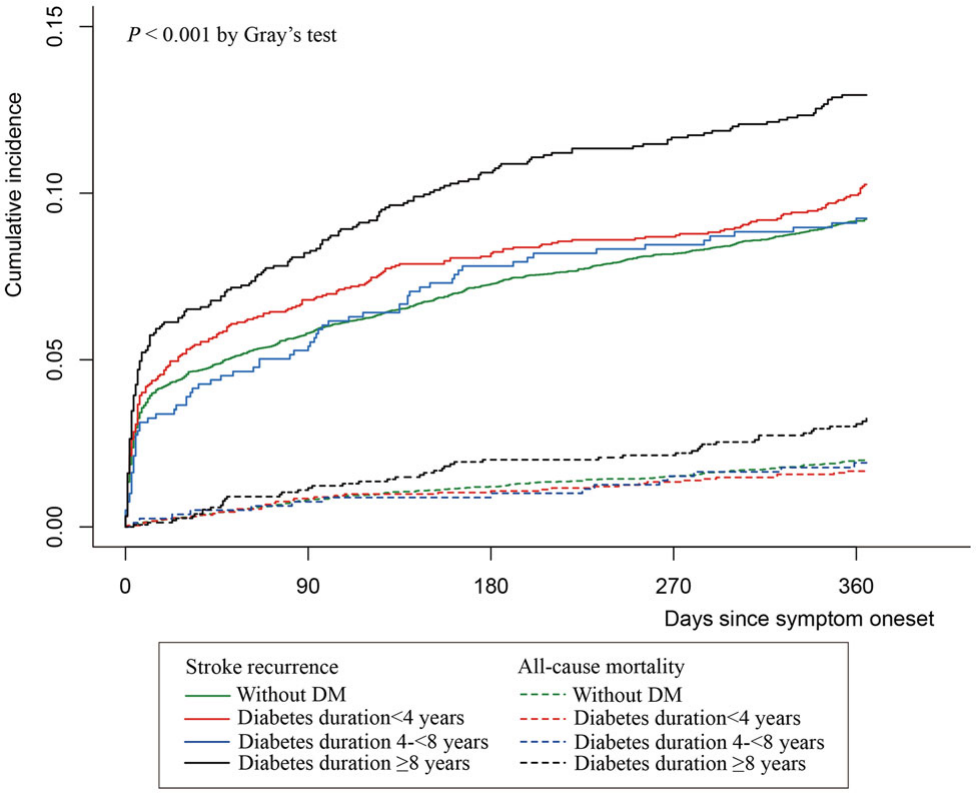
Nelson‒Aalen cumulative hazard estimates of stroke recurrence and all‐cause mortality. Abbreviation: DM, diabetes mellitus

**FIGURE 3:**
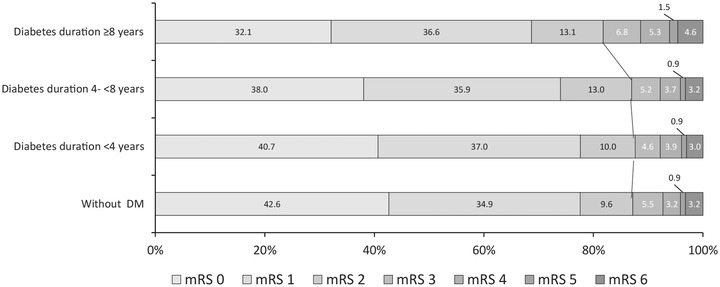
Distribution of mRS scores at 12 months among different diabetes duration groups. Abbreviations: mRS, modified Rankin Scale; DM, diabetes mellitus

**FIGURE 4:**
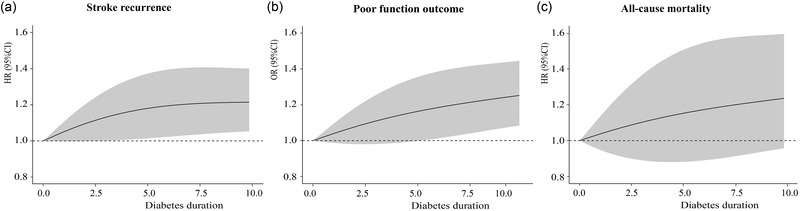
Restricted cubic spline analysis between diabetes duration and risks of stroke recurrence (a), poor function outcome (b), and all‐cause mortality (c). Abbreviations: CI, confidence interval; HR, hazard ratio; OR, odds ratio

In the model adjusting for demographics of age and sex (model 1), we found that diabetes duration ≥8 years was associated with an increased risk of 1‐year stroke recurrence (HR, 1.40; 95% CI, 1.20–1.63; *p *< .001) and poor function outcome (OR, 1.37; 95% CI, 1.18–1.59; *p *< .001) compared with those without DM (Table 2). After adjusting for demographics, medical history, index event, stroke severity and subtype on admission, and medication at discharge (model 2), a diabetes duration ≥8 years was only associated with an increased risk of stroke recurrence (HR, 1.31; 95% CI, 1.05–1.64; *p *= .02). In model 3 with additional adjustment for HbA1c, patients with a diabetes duration ≥8 years were associated with 1‐year stroke recurrence (HR, 1.25; 95% CI, 1.00–1.55; *p *< .052) and all‐cause mortality (HR, 1.66; 95% CI, 1.09–2.52; *p *= .02). All of the proportional hazard assumptions were met (*P *> 0.05). Using a competing risk regression model, a diabetes duration ≥8 years was a significant risk factor for stroke recurrence (HR, 1.31; 95% CI, 1.12–1.53). *p*‐Values for trends used to assess the association with clinical outcomes, including stroke recurrence, poor function outcome, and all‐cause mortality, were also not significant. The Nelson Aalen estimation of the cumulative incidence of stroke recurrence and all‐cause mortality are shown in Figure 2. Figure 3 shows the distribution of mRS scores at the 1‐year follow‐up in different diabetes duration groups. The restricted cubic spline analysis revealed that a longer diabetes duration was associated with a higher risk of stroke recurrence (Figure 4).

**TABLE 2:** Associations between diabetes duration and stroke prognosis

	Groups	Events, *N* (%)	Model 1^a^		Model 2^b^		Model 3^c^	
			HR/OR (95% CI)^d^	*p*‐Value	HR/OR (95% CI)	*p*‐Value	HR/OR (95% CI)	*p*‐Value
Stroke recurrence	Without DM	920 (9.1)	Ref.		Ref.		Ref.	
	Diabetes duration <4 years	227 (10.1)	1.13 (0.97–1.30)	.11	1.11 (0.92–1.33)	.28	1.09 (0.92–1.30)	.33
	Diabetes duration 4 to <8 years	73 (9.2)	1.00 (0.79–1.27)	.99	0.97 (0.73–1.29)	.82	0.91 (0.68–1.21)	.51
	Diabetes duration ≥8 years	199 (12.8)	1.40 (1.20–1.63)	<.001	1.31 (1.05–1.64)	.02	1.25 (1.00–1.55)	.052
Poor function outcome|^e^	Without DM	1263 (12.9)	Ref.		Ref.		Ref.	
	Diabetes duration <4 years	273 (12.4)	1.02 (0.89–1.18)	.77	0.95 (0.78–1.15)	.58	0.94 (0.77–1.14)	.50
	Diabetes duration 4 to <8 years	102 (13.0)	1.04 (0.83–1.30)	.73	0.92 (0.69–1.23)	.56	0.89 (0.66–1.21)	.46
	Diabetes duration ≥8 years	274 (18.2)	1.37 (1.18–1.59)	<.001	1.20 (0.95–1.51)	.14	1.19 (0.94–1.52)	.15
All‐cause mortality	Without DM	318 (3.2)	Ref.		Ref.		Ref.	
	Diabetes duration <4 years	66 (3.0)	1.03 (0.79–1.35)	.82	1.21 (0.87–1.67)	.26	1.66 (1.21–2.27)	.002
	Diabetes duration 4 to <8 years	25 (3.1)	1.05 (0.70–1.57)	.83	1.22 (0.74–2.01)	.43	1.90 (1.17–3.08)	.009
	Diabetes duration ≥8 years	69 (4.5)	1.29 (1.00–1.68)	.051	1.46 (1.00–2.13)	.050	1.66 (1.09–2.52)	.02

Abbreviations: CI, confidence interval; DM, diabetes mellitus; HR, hazard ratio; OR, odds ratio.

^a^ Model 1: adjusted for age and sex.

^b^ Model 2: adjusted for model 1 plus current smoking, regular drinking, prior stroke, hypertension, dyslipidemia, atrial fibrillation, coronary artery disease, index event, stroke subtype defined by the Trial of Org 10172 in Acute Stroke Treatment classification, National Institutes of Health stroke scale, estimated glomerular filtration rate on admission, antihypertensive drugs, lipid‐lowering drugs, antiplatelet agents, and hypoglycemic therapy for discharge.

^c^ Model 3: adjusted for model 2 plus inverse probability‐weighted analysis for missing data of hemoglobin A1c.

^d^ The Cox proportional hazard regression model was used to assess the variables of stroke recurrence and all‐cause mortality from 1 year by calculating HRs and 95% CIs. The multivariable logistic regression model was used for the assessment of variables that were associated with poor functional outcome by calculating ORs and 95% CIs.

^|e^ Poor function outcome refers to modified Rankin Scale 3–6.

## 4 DISCUSSION

From the large‐scale stroke registry in China, our study demonstrated that long‐term diabetes duration (≥8 years), but not short‐term diabetes duration, was associated with an increased risk of stroke recurrence in Chinese patients, which was independent of HbA1c at baseline and other traditional risk factors.

A wide range of available published data have demonstrated that diabetes duration is a predictor of cardiovascular disease and mortality (Noh et al., 2017; Li et al., 2021). Subsequently, the association between diabetes duration and ischemic stroke was also published in the Northern Manhattan Study (Banerjee et al., 2012). It has been observed in previous works that risks of cardiovascular disease and mortality are strongly associated with diabetes increased with an increasing diabetes duration in comparison to patients without DM (Arredondo, 2018; Li et al., 2021). Moreover, a study from the Mexico population (Gao et al., 2017) showed that diabetes duration was a prognostic factor for major adverse cardiac or cerebrovascular events. Another previous study published by the Ludwigshafen Risk and Cardiovascular Health Study showed that long‐term type 2 diabetes was significantly associated with coronary heart disease, cerebrovascular disease, and peripheral vascular disease (Silbernagel et al., 2012), which indicated that diabetes duration was a determinant of vascular disease. Moreover, a few studies have also observed an association between newly diagnosed DM and poor prognosis at 1 year after ischemic stroke (Jing et al., 2016). Importantly, broad evidence has shown that diabetes duration is associated with an increased risk of stroke (Air & Kissela, 2007; Banerjee et al., 2012; Tseng et al., 2005). Certain predictors of stroke, such as type 2 DM, were found to be time‐specific and may differ in late and early recurrences (Feigin et al., 2016; Y. J. Wang et al., 2020). In summary, these studies less focused on the influence of diabetes duration on the prognosis of stroke. Our study further demonstrated that long‐term diabetes duration (≥8 years) was associated with a higher risk of 1‐year stroke recurrence even when HbA1c and other confounders were controlled. This indicates that the status of DM per se was of clinical significance, independent of the control status of the blood glucose level.

Several potential mechanisms linking long‐term diabetes duration and stroke recurrence have been proposed. First, long‐term diabetic patients featured by insufficient insulin secretion weakens endothelial cell function and inhibits the generation of nitric oxide (NO) in comparison to short‐term diabetes patients mainly characterized by insulin resistance. This observation suggests that factors such as aberrant HbA1c and endothelial NO level in patients with long‐term diabetes duration leading to irreversible endothelial dysfunction and impaired vascular tone are responsible for stroke recurrence (Nazir et al., 2006; Petrie et al., 1996). Second, several mechanisms, such as excess production of oxidative stress, activation of protein kinase C, and receptor for advanced glycation end products (Novak et al., 2011; Novak et al., 2006), account for disruptions of microvascular function, which thus accelerate atherosclerosis and plaque progression. With accumulation of vascular damage over time, it further leads to trigger the occurrence of stroke recurrence. Third, the risk of microalbuminuria has been reported to be increased with increasing diabetes duration (Tuomilehto et al., 1998; D. Wang et al., 2021), and microalbuminuria has been proven to be an independent risk factor for stroke in diabetic patients (Guerrero‐Romero & Rodríguez‐Morán, 1999). Furthermore, impairment in fibrinogen and clotting mechanisms with long‐term diabetes duration also contributes to pathological reasons for stroke recurrence (Kannel et al., 1990; Meigs et al., 2000).

Our study still has some limitations. First, owing to the missing data of the HbA1c index, the association between diabetes duration and stroke recurrence may be biased. However, after using inverse probability‐weighted analysis to limit the influence of missing data in model 3, we found similar results. Second, since diabetes duration at baseline based on the calculation of patients’ self‐reported age at diabetes onset may be inaccurate, a lag time between diabetes onset and diagnosis exists (Harris et al., 1992). There may be several years of pathoglycemia before diabetes diagnosis, which also increases the risk of stroke recurrence. Third, new onset DM after discharge was not recorded in this study. Finally, a portion of high‐risk patients with ischemic stroke or TIA might be missed due to being recruited within 7 days. Consequently, the existence of misclassification bias is inevitable. This finding needs to be further verified in future studies with large populations.

## 5 CONCLUSIONS

In this national registry of ischemic stroke or transient ischemic attack patients in China, long‐term diabetes duration, but not short‐term diabetes duration, was associated with an increased risk of 1‐year stroke recurrence. Future studies should focus on how to control and prevent stroke recurrence in patients with long‐term diabetes duration.

## CONFLICT OF INTEREST

None.

### PEER REVIEW

The peer review history for this article is available at: https://publons.com/publon/10.1002/brb3.2725.

## Data Availability

Data are available to researchers on request for purposes of reproducing the results by directly contacting the corresponding author.
